# A colorimetric readout based on the PrestoBlue® dye as an objective method for measuring neutralizing antibodies against influenza virus in human serum samples

**DOI:** 10.1016/j.bbrep.2026.102639

**Published:** 2026-06-22

**Authors:** Maria A. Stincarelli, Francesca Dapporto, Valentina Biagini, Maria Giovanna Marotta, Stefano Gonzi, Alessandro Manenti, Emanuele Montomoli

**Affiliations:** a VisMederi Srl, Siena, Italy; b Department of Life Sciences, University of Siena, Siena, Italy; c Department of Molecular and Developmental Medicine, University of Siena, Siena, Italy

## Abstract

Influenza is an infectious respiratory disease that has significant morbidity and a high mortality rate. The immune responses induced against both seasonal and potentially pandemic strains of the influenza virus are of fundamental importance. The microneutralization (MN) assay is an extremely important virological test for the evaluation of vaccines, and therefore of the antibody response. However, there is not always a correspondence between the results obtained from the same samples, owing to the difficult readout of the cytopathic effect (CPE) in the case of live viruses and the subjectivity of interpretation by operators. Moreover, if the test is combined with the Enzyme-Linked Immunosorbent Assay (ELISA) technique, the process is laborious. Here, we present a microneutralization assay that uses the PrestoBlue® reagent as a colorimetric CPE-based method. We evaluated the performance of this test on multiple strains of influenza virus by using human serum samples from a commercial panel and comparing both subjective (inverted microscope) and objective (spectrophotometer) readouts. Our results suggest that this colorimetric method for assessing CPE in microneutralization tests is more sensitive than the subjective method, while yielding comparable titers. This assay allows accurate and repeatable reading of the results, thereby reducing the possible uncertainty of the readout and speeding up the process.

## Highlights

• Influenza is a respiratory disease with significant morbidity and mortality rate.
• The microneutralization assay is an important virological test for vaccines evaluation.
• There is not always a correspondence between results obtained from the same samples.
• PrestoBlue® method for assessing CPE in microneutralization tests is more sensitive.
• This assay reduces the uncertainty of the readout and speeds up the process.

## Graphical abstract

**Figure:**
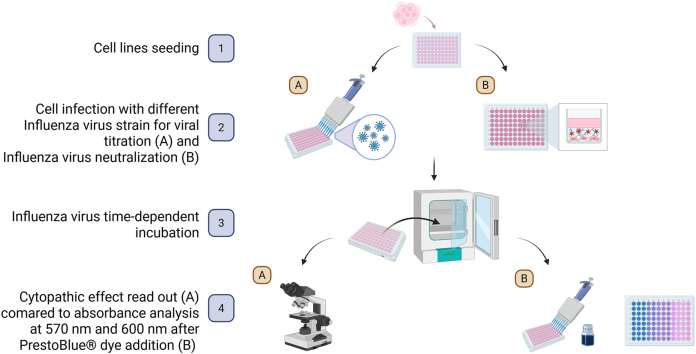

## 1 Introduction

Influenza viruses are segmented, negative-sense RNA viruses belonging to the *Orthomyxoviridae* family. They constitute a public health concern owing to their capacity to cause seasonal epidemics and occasional pandemics [1]. Influenza viruses are classified into four types: A, B, C and D. Among these, human influenza viruses A and B are primarily responsible for seasonal outbreaks, with influenza A viruses posing the greatest pandemic threat, owing to their genetic variability and zoonotic potential [2,3]. The continuous evolution of these viruses, owing to their ability to undergo antigenic drift and shift, complicates long-term vaccine efficacy and demands ongoing surveillance and vaccine reformulation.

Seasonal human influenza A strains (IAV), such as H1N1 and H3N2 subtypes, are responsible for up to 5 million cases of severe illness annually, causing 290,000 to 650,000 deaths worldwide, according to the World Health Organization (WHO) [1,3]. Surveillance systems coordinated by the WHO, such as the Global Initiative on Sharing All Influenza Data (GISAID) and the Global Influenza Surveillance and Response System (GISRS), monitor circulating strains in order to inform vaccine composition and detect emerging variants with pandemic potential. The antigenic drift of influenza viruses necessitates the reformulation of seasonal vaccines, typically quadrivalent formulations containing two influenza A subtypes (H1N1 and H3N2) and two influenza B lineages (Victoria and Yamagata) [4].

In recent years, the emergence and spread of highly pathogenic avian influenza (HPAI) viruses, particularly the H5N1 subtype, have heightened concerns about zoonotic transmission and pandemic risk [4,5]. Since its initial detection in 1997, its reemergence in 2003 and outbreak in birds in 2014, H5N1 has displayed high mortality rates in animals and sporadic human infections, these latter often being linked to close contact with infected poultry and cows [5]. The constant circulation of H5N1 in poultry and wild birds increases the risk of genetic changes that could facilitate transmission to humans [6]. Although sustained human-to-human transmission has not yet occurred, the genetic adaptability of the virus underscores the need for robust epidemiological monitoring and preparedness strategies.

Current research is focusing on improving vaccine platforms, including the development of cell-based, recombinant, and mRNA vaccines, which offer advantages in terms of production speed and antigenic match [[7], [8], [9], [10]]. Additionally, many efforts are underway to develop universal influenza vaccines that target conserved viral epitopes, in order to provide broader and longer-lasting protection across subtypes and strains, including zoonotic viruses like H5N1 [11,12].

Neutralization assays are essential tools for evaluating immune responses to influenza infection and vaccination and offer functional insight into antibody-mediated protection [13,14]. Among these assays, the most widely used are the hemagglutination inhibition (HAI) assay, the microneutralization (MN) assay, and the enzyme-linked immunosorbent–microneutralization (ELISA-MN) assay [[15], [16], [17]]. Traditionally regarded as the standard for evaluating influenza vaccine responses, the HAI assay quantifies antibodies that prevent viral hemagglutinin from binding to red blood cells [17,18]. Although this assay is rapid and cost-effective, its utility is limited by its low sensitivity to certain subtypes and its reliance on virus-specific receptor-binding properties [19,20]. Moreover, the sensitivity of the HAI assay is strictly dependent on the source of red blood cells (RBCs), which hinders standardization among laboratories. The MN assay, which is based on the evaluation of cytopathic effect (CPE), is extremely sensitive and suitable for detecting functional neutralizing antibodies against a broad range of influenza viruses, including those with limited hemagglutination activity, such as avian strains like H5N1 [17,21,22]. It is particularly valuable in vaccine efficacy studies and in assessing the cross-protection induced by candidate vaccines.

The ELISA-MN assay combines the specificity of ELISA with the functional readout of virus neutralization, thereby enabling high-throughput screening and enhanced quantification of neutralizing antibodies. However, these antibodies are mainly involved in the first phase of the viral cycle (attachment/entry), bypassing those involved in later stages of infection (e.g., anti-NA), which are, by contrast, detected by means of CPE-based methods. This makes it especially useful in large-scale sero-epidemiological studies and in assessing responses to next-generation or pandemic candidate vaccines [23].

Together, these methods form a powerful tool for analyzing the immunological protection provided by vaccine immunization. However, the high cost of reagents and the difficulty of interpretation, especially with CPE-based methods, require significant resources and highly specialized personnel [22,23]. In order to overcome these problems, it is necessary to develop alternative methods based on objective readouts. Thus, the aim of this study was to demonstrate the objectivity of PrestoBlue® as a colorimetric method for a high-throughput microneutralization test for the detection of neutralizing antibodies (nAbs) against different influenza strains in human serum samples.

## 2 Materials and methods

### 2.1 Cell and virus propagation

Madin-Darby Canine Kidney (MDCK) cells (ATCC CCL-34) were grown in Eagle's Minimum Essential Medium (EMEM) (Euroclone) supplemented with 5% v/v fetal bovine serum (FBS) (Euroclone), 2 mM l-glutamine, 100 units per mL penicillin and 100 μg per mL streptomycin (P/S) (Gibco). Cells were maintained at 37 °C in a humified 5% CO_2_ environment and passed every 3-4 days.

MDCK-SIAT1 (Francis Crick Institute, Cat #: P4), derived from the stable transfection of MDCK cells with the cDNA of human 2,6-sialtransferase (SIAT1), were grown in Dulbecco's Modified Eagle's Medium (DMEM) (Euroclone) supplemented with 10% v/v FBS, 2 mM l-glutamine, 200 units per mL geneticin (Gibco) and 1% Non-Essential Amino Acids NEAA (Gibco). Cells were maintained at 37 °C in a humified 5% CO_2_ environment and passed every 2-3 days.

The influenza viruses strain used were: A/Cambodia/e0826360/2020 (H3N2) (National Institute for Biological Standards and Control, NIBSC, code 30/112), A/Wisconsin/588/2019 (H1N1), B/Phuket/3073/2013 (B Yamagata), B/Washington/02/2019 (B Victoria) (The Francis Crick Institute), and A/Texas/37/202 (H5N1) (Centers for Disease Control and Prevention, CDC).

For each strain, the virus was grown in T175 tissue-culture flasks pre-seeded with MDCK cells at a concentration of 1.3 × 10^5^ cells per mL in EMEM supplemented 0.5% FBS. Briefly, after 24 h of incubation at 37 °C with 5% CO_2_, flasks were washed twice with Dulbecco's phosphate buffered saline (DPBS) (Gibco) and infected at a multiplicity of infection (MOI) of 0.001 in OptiPro™ (Gibco) supplemented with 2 mM l-glutamine, 100 units/mL penicillin and 100 μg/ml streptomycin and 5 μg per ml acetylated trypsin (Sigma-Aldrich). Flasks were infected and incubated for 1 h at 37 °C or 35 °C with 5% CO_2_ for influenza A and B types, respectively, then filled with complete OptiPro™ supplemented with 11 μg per ml acetylated trypsin. Cells were harvested when the flasks reached 70–80% of CPE, and the supernatant was centrifuged at 296 g for 5 min at 4 °C. The viral solution was then aliquoted and stored at −80 °C.

### 2.2 Serum samples

A total of 16 commercially available human serum samples (Golden West, SD1060) were screened for H1N1, H3N2, B/Victoria and B/Yamagata strains. Serum samples were heat-inactivated by means of incubation at 56 °C for 30 min before testing. The samples were also treated with the receptor-destroying enzyme (RDE) (Denka Seiken) according to the manufacturer's instructions. Briefly, one volume of a serum sample was added to three volumes of RDE and incubated overnight at 37 °C. Sera were then incubated at 56 °C for 1 h to inactivate the enzyme.

### 2.3 Viral titration

Viruses were titrated in log-1 serial dilutions (from log-1 to log-11) to obtain a 50% tissue culture infective dose (TCID_50_) on 96‐well culture plates of MDCK and MDCK-SIAT1 cells. Plates were recorded daily until day 7. The end‐point titers were calculated according to the Reed & Muench method based on eight replicates per titration, expressed as log_10_ TCID_50_ per mL.

### 2.4 Microneutralization assay

Sixteen serum samples were evaluated for nAbs against all the influenza virus strain. After two‐fold serial dilution, starting from 1:10, samples were mixed with an equal volume of viral solution containing 100 TCID_50_ of the respective influenza virus strain. The serum‐virus mixture was then incubated for 1 h at 37 °C with 5% CO_2_. After incubation, 90 μL of the mixture at each dilution was added in duplicate to a cell plate containing 300,000 cells/mL confluent MDCK monolayer supplemented with 11 μg/mL acetylated trypsin. The plates were incubated for 7 days at 37 °C with 5% CO_2_.

After 5 to 7 days of incubation, plates were read with an inverted optical microscope. The highest serum dilution that protected more than 50% of cells from CPE was taken as the neutralization titer. Then, 10 μL of PrestoBlue® solution (Life Technologies) was added to the supernatant of each well of the MN plates. After 2 h of incubation at 37 °C with 5% CO_2_, plates were read by means of a spectrophotometer set at 570 nm and 600 nm wavelengths, according to the manufacturer's instructions. The reciprocal of the highest serum dilution that yielded an optical density (OD) value greater than the cut‐off value was taken as the neutralization titer. The cut‐off value was calculated as the average of the OD values of the viral control wells subtracted from the average of the OD values of the control cells and divided by two.

### 2.5 Statistical analysis

Data analysis was performed by means of GraphPad Prism software version 10.2.3. Spearman's rank correlation coefficient analysis was used to compare MN-CPE and MN-PrestoBlue® antibody titers. A P value < 0.05 was considered statistically significant.

## 3 Results

### 3.1 Optimization of cell seeding and pre-incubation time

To assess the viral titer of each influenza virus strain used, it was first necessary to determine the number of cells per well and the pre-incubation time before infection. Cell densities of 150,000 cells/mL, 200,000 cells/mL and 300,000 cells/mL were evaluated in combination with two pre-incubation times of 24 and 48 h. The selection of these conditions was aimed at determining the best condition in which to obtain the formation of a confluent cell monolayer and to ensure cell viability at the time of infection.

The viral titer was calculated on using both the CPE-based method and the colorimetric readout in PrestoBlue® 7 days after infection (Table 1); in this latter case, the PrestoBlue® solution was added to the well and the reduction from resazurin to resofurin was measured by means of a spectrophotometer. On applying the CPE-based method, the H1N1 A/Wisconsin strain in plates pre-seeded at 24 h showed the highest titer (average titer) at a cell density of 150,000 cells per mL, while with the PrestoBlue® readout the highest titer (average titer) was reached at 200,000 cells per mL. In contrast, in plates pre-seeded at 48 h, the highest titer (average titer) was reached at 300,000 cells per mL with PrestoBlue®. The H3N2 A/Cambodia strain, on the other hand, showed a similar titer in each condition tested, reaching a titer of 6.39 Log_10_ TCID50/mL with the CPE-based method and 4.73 Log_10_ TCID50/mL with the PrestoBlue® readout, on using 300,000 cells/mL with 48-h pre-seed. Regarding the B/Washington Victoria-lineage strain, higher titers were achieved (average titer) on using a cell density of 300,000 cells/mL, and the best titer was reached with a 48-h pre-seed (12.54 Log_10_ TCID50/mL for both 200,000 cell/mL and 300,000 cells/mL with PrestoBlue®). The B/Phuket Yamagata-lineage strain reached a titer of 10.57 Log_10_ TCID50/mL at a cell density of 200,000 cells/mL in 24-h pre-seeded plates, while with 48-h pre-seeding the maximum titer was 11.50 Log_10_ TCID50/mL at a cell density of 300,000 cells/mL.

**Table 1:** Comparison of log_10_ TCID_50_ viral titers obtained at different cell density concentrations after 24-h and 48-h pre-seed.

Strain	Days Post-Infection (DPI)	24 h pre-seed (cells per mL)			48 h pre-seed (cells per mL)		
		150,000	200,000	300,000	150,000	200,000	300,000
**A/Wisconsin**	5	7.48	6.81	6.80	7.80	7.60	7.55
	6	7.59	7.00	6.80	7.90	7.81	7.66
	7	7.59	7.00	6.80	7.90	7.91	7.70
	**7 PB**^a^	**8.86**	**9.00**	**6.58**	**8.00**	**8.71**	**9.36**
**A/Cambodia**	5	5.57	5.23	5.49	5.50	5.21	5.75
	6	5.57	5.23	5.49	5.59	5.50	5.96
	7	5.57	5.23	5.49	5.59	5.85	6.39
	**7 PB**	**4.57**	**4.43**	**4.59**	**4.75**	**4.79**	**4.73**
**B/Washington**	5	7.57	7.04	7.50	9.07	9.58	9.63
	6	8.39	8.41	8.50	9.21	9.71	9.71
	7	8.39	8.41	8.50	9.21	9.78	9.71
	**7 PB**	**9.45**	**8.59**	**10.05**	**11.75**	**12.54**	**12.54**
**B/Phuket**	5	7.66	7.89	7.25	9.00	8.59	9.00
	6	8.20	8.22	8.00	9.54	9.20	9.22
	7	8.20	8.22	8.00	9.54	9.20	9.22
	**7 PB**	**9.43**	**10.57**	**8.00**	**11.22**	**11.33**	**11.50**

Log_10_ TCID_50_ values are expressed as the mean of three independent experiments.

a PB, PrestoBlue® staining.

### 3.2 Comparison between CPE-based and PrestoBlue®-based MN assays

A total of 16 serum samples from a commercial panel were tested for the presence of anti‐influenza virus antibodies by means of the CPE-based and PrestoBlue® MN assays. Results of the comparison between these two assays (Table 2) showed that a well‐trained operator was able to read the CPE, although the titers were more conservative owing to the subjectivity of the reading method; indeed, the titers read in PrestoBlue® were higher than those obtained with the CPE readout. However, since the cut-off was calculated mathematically, the titers yielded by CPE represent 50% of the cell monolayer in comparison with the control cells. Nevertheless, the same trend was maintained for both CPE and PrestoBlue® readings, with titers higher than 2-fold being observed for the two type A influenza viruses, particularly A/Wisconsin. Among type B influenza viruses, titers were higher for B/Phuket.

**Table 2:** Titers against influenza viruses on micro-neutralization.

Sample ID	A/Wisconsin		A/Cambodia		B/Washington		B/Phuket	
	CPE	PrestoBlue®	CPE	PrestoBlue®	CPE	PrestoBlue®	CPE	PrestoBlue®
W425623103256	55	320	320	905	196	320	196	554
W425522110155	80	80	320	1280	196	480	240	554
W425523107072	495	2217	320	905	89	392	620	1280
W425623000201	975	2862	320	1280	15	160	80	554
W425623000387	1340	3620	453	1810	139	320	1239	3840
W425623102938	1518	3620	453	1280	139	453	905	4434
W425623103247	3684	3620	226	640	49	392	69	277
W425623103272	1043	3135	320	905	24	200	196	554
W425623105197	277	453	226	320	71	277	219	960
W425623105567	320	453	453	1280	69	226	139	679
W425623105575	620	1109	640	2560	120	339	294	960
W425623105581	509	1810	320	1280	160	453	320	905
W425623105590	1979	5120	640	1280	20	160	226	453
W425623105600	759	1109	320	1280	160	320	1810	5120
W425623105609	1320	3620	320	640	28	160	905	5120
W425623105610	1126	1280	160	320	20	80	80	160

Values of titers are expressed as the geometric mean of three independent experiments.

### 3.3 RDE treatment of serum samples

**Fig. 1:**
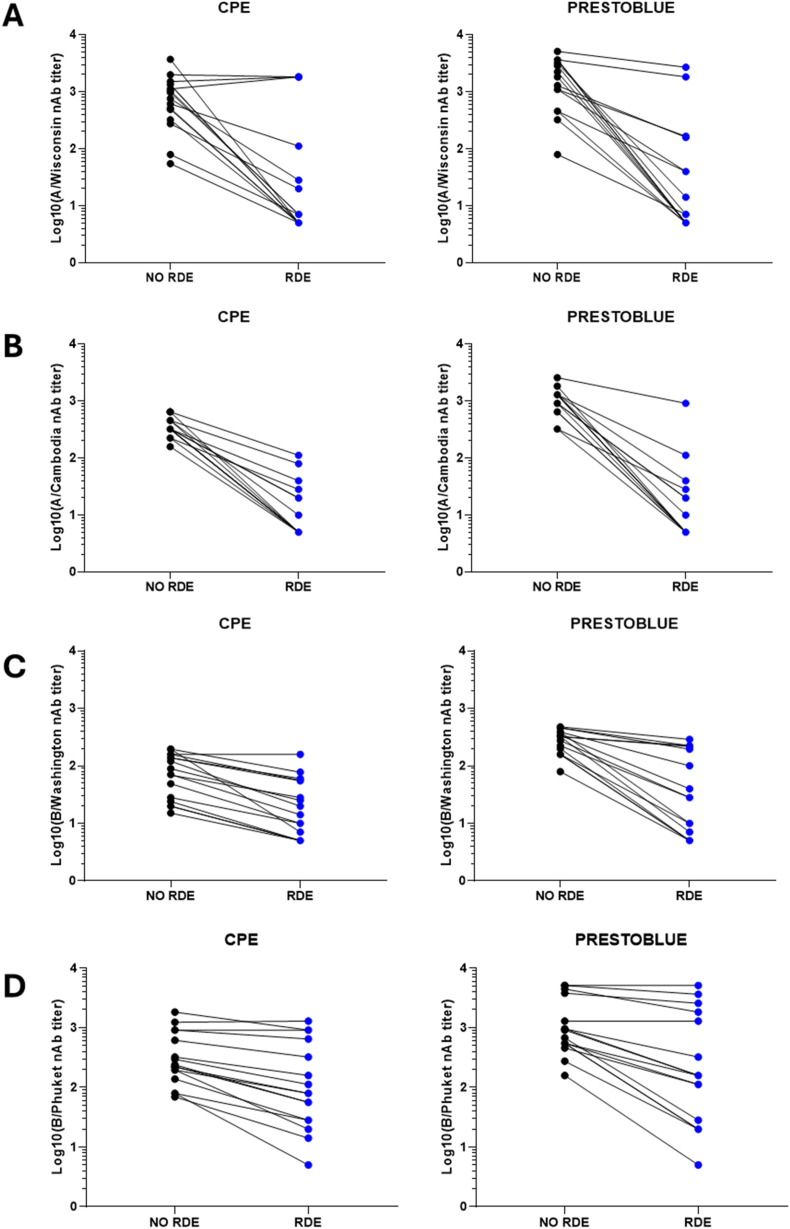
Comparison between the CPE- and PrestoBlue®-MN assays, with and without RDE treatment. Paired graphs comparing antibody titers derived from CPE and PrestoBlue® readout, with and without RDE treatment of samples; each dot represents the GMT expressed in log_10_ of a single serum sample tested in triplicate by means of each method for A/Wisconsin/588/2019 (H1N1). (**A**), A/Cambodia/e0826360/2020 (H3N2) (**B**), B/Washington/02/2019 (B Victoria) (**C**), and B/Phuket/3073/2013 (B Yamagata) (**D**). Differences between RDE-treated and untreated samples were assessed using a paired *t*-test on log_10_ transformed GMTs. The corresponding p-values, reflecting the statistical significance of these differences, are reported for both CPE and PrestoBlue® readouts for each strain.

**Fig. 2:**
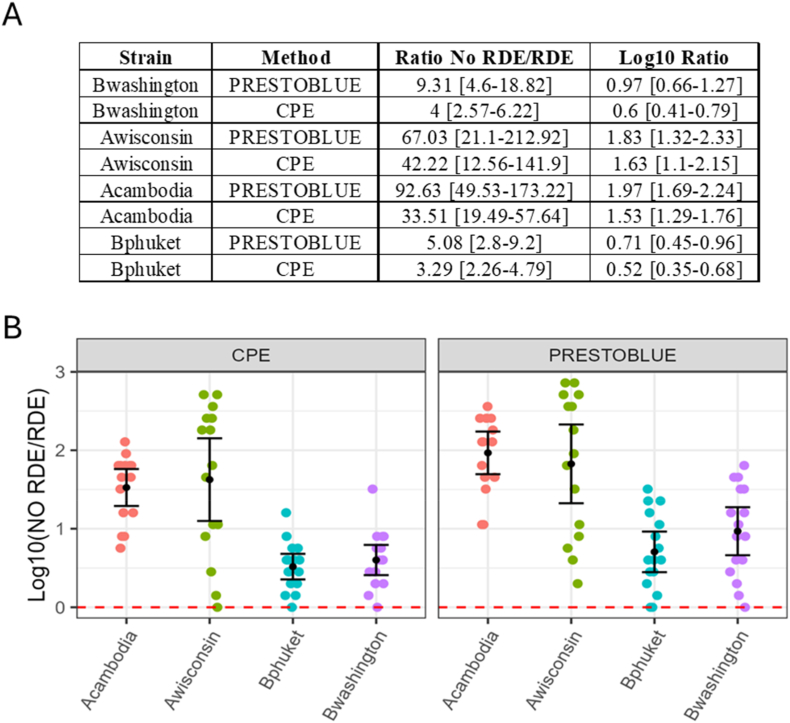
Fold differences analysis between CPE and PrestoBlue® readouts for influenza A and B strains. Table (A) reports, for each strain, the mean ratio between RDE-treated and untreated samples (RDE/No RDE) for both PrestoBlue® and CPE readouts, together with their 95% confidence intervals, shown both on the linear and log10 scales. The mean ratio between RDE-treated and untreated samples was calculated on the log10 scale by first computing the individual log10-transformed ratios for each sample and then averaging these values. The 95% confidence intervals were also derived from the log10-transformed ratios. Specifically, the standard error of the mean log10 ratio was calculated, and the corresponding t-statistic with (n−1) degrees of freedom was applied to define the 95% confidence intervals. The resulting lower and upper bounds, together with the mean, were then back-transformed to the linear scale to facilitate interpretation. Figure (B) displays, for each strain and each readout method, the individual RDE/No RDE ratios for all samples, along with the corresponding mean ratio and its 95% confidence interval, expressed on the log10 scale. The dashed line at 0 indicates no difference between RDE-treated and untreated samples; values above this line indicate higher values in untreated samples, whereas values below indicate higher values in RDE-treated samples.

To avoid false-positive results due to the non-specific binding of serum proteins with viral antigens, sera were pre-treated with RDE. As shown in Fig. 1, pre-treatment seems to drastically reduce antibody titers. However, antibody titers against the B/Phuket strain remained higher than those against the other three strains tested. As illustrated in Fig. 1, titers against influenza A strains were drastically reduced (panels A and B). Titers against influenza B strains displayed a similar trend (panels C and D), though they remained more stable. Specifically, pre-treatment with RDE reduced titers against A/Wisconsin on applying both the CPE method and the PrestoBlue® method (panel A), but titers for both methods were more similar after treatment. The greatest differences can be observed with regard to the A/Cambodia strain (panel B), for which the titers of untreated sera already showed significant differences between the two methods. Following treatment with RDE, however, the titers yielded by the CPE method were much lower and more homogeneous than those yielded by the colorimetric method. Panel C shows the pattern of the titers against the B/Washington strain with and without RDE treatment. As can be seen, RDE treatment reduced the difference between the titers yielded by the two methods, making the antibody titers much more similar and comparable. The B/Phuket strain (panel D), on the other hand, showed the least differences between the two readout methods, whether with or without RDE treatment. As shown in Fig. 2, for both methods the ratios between NO RDE and RDE were calculated, with mean and 95% confidence. As shown, NO RDE treatment tended to have higher values than RDE treatment for all strains, and the ratios obtained with PrestoBlue® were generally higher than those obtained with CPE.

**Fig. 3:**
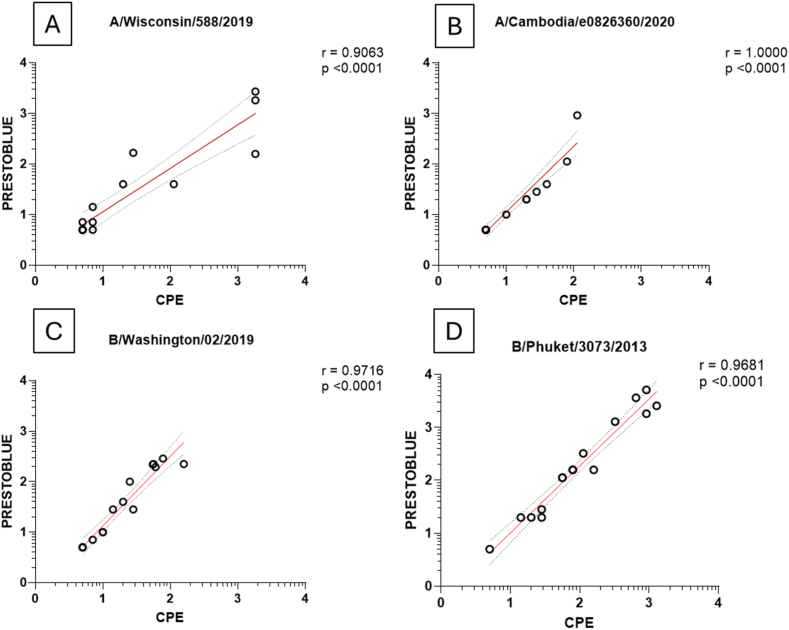
Correlation between CPE and PrestoBlue® readouts for influenza A and B strains. Spearman correlation of antibody titers yielded by CPE and PrestoBlue® readouts for A/Wisconsin/588/2019 (H1N1) (**A**), A/Cambodia/e0826360/2020 (H3N2) (**B**), B/Washington/02/2019 (B Victoria) (B Yamagata) (**C**), B/Phuket/3073/2013 (**D**). Red line indicates linear regression line and dashed lines indicate 95% confidence interval. Each dot represents the GMT expressed in log_10_ of a single serum sample tested in triplicate for each method. Spearman correlation analysis was performed to evaluate the association between CPE and PrestoBlue® readouts. For each analysis, the Spearman correlation coefficient (Rho) and the corresponding p-value were reported to assess the strength and statistical significance of the relationship.

**Fig. 4:**
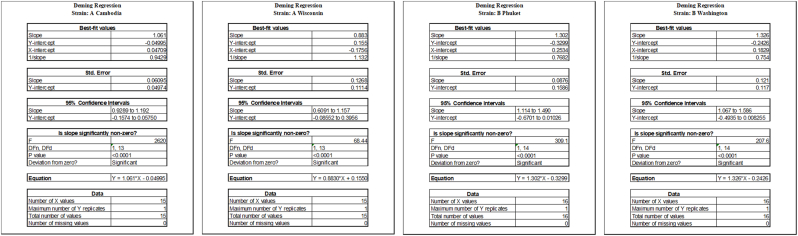
Deming regression analysis between CPE and PrestoBlue® readouts for influenza A and B strains. The tables report, for each strain, the results of the Deming regression performed on log-transformed readouts. For each analysis, the estimated slope, y-intercept, and their corresponding 95% confidence intervals are shown. Deming regression, an errors-in-variables method, is appropriate when both measurement techniques are affected by analytical error. In this analysis, the ratio of the error variances between CPE and PrestoBlue® was assumed to be equal (R = 1), implying comparable measurement variability. An ideal slope close to 1 indicates absence of proportional bias, meaning neither method systematically overestimates nor underestimates the other.

After comparing the two readout methods individually, we measured the correlation between the results yielded by both by calculating Spearman's equation. As shown in Fig. 3, a correlation emerged between the antibody titers yielded by the CPE method and those yielded by the PrestoBlue® method. Regarding the A/Cambodia strain, Spearman's rho value (r) showed a significant correlation (r = 1.0000), while the rho values for the B/Washington and B/Phuket strains were 0.9716 and 0.9681, respectively. By contrast, a moderate correlation between the two methods was observed with regard to the A/Wisconsin strain (r = 0.9063). To support this correlation, the Deming regression between the two methods was calculated and is shown in Fig. 4. Compared to classic linear regression, it considers error in both measurements. The most relevant parameters are the slope and its confidence intervals, which describe how much Prestoblue varies as CPE varies.

### 3.4 Optimization of the H3N2 influenza virus strain in MDCK-SIAT1 cell line

Since H3N2 influenza strains grow better and more stably in MDCK-SIAT1 cells, preliminary experiments were conducted to determine the appropriate cell concentration for the growth and titration of these strains. Since this cell line grows much faster than normal MDCK cells, the concentration per well was drastically reduced to 30,000, 25,000, 20,000, and 10,000 cells per mL with 24-h pre-seed. The readout was maintained at 7 days post-infection for both the CPE and PrestoBlue® methods. The data obtained from the titrations performed on the various cell concentrations are shown in Table 3; at the four cell densities used, titers were much higher than those obtained on using the normal MDCK cell line (Table 1). As the number of cells increased, so did the titer, reaching a value of 13.17 TCID_50_/mL on titration at a density of 25,000 cells per mL.

**Table 3:** Comparison of log_10_ TCID_50_ viral titers obtained in MDCK-SIAT1 cell line, from different cell density concentrations after 24-h pre-seed.

Strain	Days Post-Infection (DPI)	24-h pre-seed (cells per mL)			
		10,000	20,000	25,000	30,000
**A/Cambodia**	7	9.78	8.90	11.87	11.84
	**7 PB**^a^	**9.90**	**9.10**	**13.17**	**12.29**

Log_10_ TCID_50_ values are expressed as the mean of three independent experiments.

a PB, PrestoBlue® staining.

### 3.5 Enforceability of PrestoBlue® readout for avian H5N1 influenza virus

To confirm the reliability of the PrestoBlue® method for influenza strains other than human ones, preliminary tests were also conducted on a wild-type strain of H5N1. As this is a very aggressive strain, its CPE was evaluated by starting at a concentration of 150,000 cells per mL with 24-h pre-seeding and monitoring the cytopathic effect already 72 h after infection. Five days post-infection, the CPE remained stable and PrestoBlue® was then added (Table 4). In this case, however, the dye incubation time was drastically reduced to as little as 40 min after incubation (data not shown).

**Table 4:** Comparison of log_10_ TCID_50_ viral titers obtained from A/Texas/37/202 (H5N1) strain on different days post-infection.

Strain	Days Post- Infection (DPI)	150,000 cells per mL	PrestoBlue®
**A/Texas**	3	16.81	an/a^a^
	4	17.00	n/a
	5	17.41	17.25
	6	17.41	17.25
	7	17.41	17.25

Log_10_ TCID_50_ values are expressed as the mean of three independent experiments.

a n/a, not analyzed.

## 4 Discussion

**Fig. 5:**
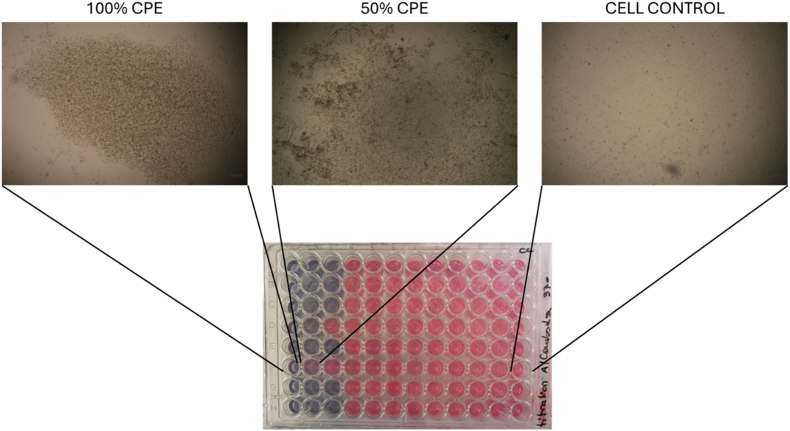
Example of CPE and PrestoBlue® readout. Visual representation of correspondence between different percentage (100%, 50% and 0% - cell control) of cytopathic effect and PrestoBlue®. The blue colored wells represent the un-reduced PrestoBlue® indicating the completely death of the cellular monolayer. The 50% CPE shows a partial reduction of the compound resulting in a purple staining of the well. Cell control, due to its un-altered monolayer, is capable of the completely reduction of the PrestoBlue® resulting in a bright pink staining.

In this study, we implemented a colorimetric method based on the use of PrestoBlue® in order to evaluate the neutralizing capacity of antibodies against various strains of influenza virus. A representative image of differences between CPE and PrestoBlue® methods is shown in Fig. 5. Our optimization of the method first concerned cell concentration, since the right balance between the number of cells tested and the virus used would guarantee not only a reliable readout but also good functionality of the PrestoBlue® dye. As viable cells can reduce resazurin to resofurin through mitochondrial activity, only those that are still viable and functionally efficient are able to change the dye from blue to red. The best condition for MDCK cell seeding was found to be 300,000 cells/mL with 48-h pre-seeding. This was not unexpected, as influenza strains, in addition to their cytopathic capacity, require a confluent cell layer to infect that must be resistant to the trypsin used in the medium. In tests performed at various cell concentrations, the colorimetric method yielded higher titers than the CPE method. PrestoBlue® is an objective method based on a mathematically calculated cut-off that considers control cells and precisely establishes 50% of the cytopathic effect. This datum cannot be obtained by observing CPE alone, and, even if the operator performing the analysis is highly qualified, it is preferable to keep the data as stringent as possible, in order to avoid overestimating the viral concentration.

Once the appropriate cell concentration and viral titer had been established, we performed microneutralization assays by using 16 sera from a commercial panel. Although the CPE method is more conservative than the colorimetric method, the trend remained unchanged. It is interesting that, according to both methods, the antibody titers against the A/Wisconsin strain were higher than those against any of the other strains tested. The seasonal influenza vaccine formulation involves the administration of a trivalent or quadrivalent vaccine derived from embryonated chicken eggs or cell cultures/recombinants. As the A/Wisconsin strain is one of the strains used in trivalent formulations, a high antibody titer against this virus is to be expected. In addition, H1N1 strains have less variability than H3N2 strains, making H1N1 strains more stable and the vaccine more effective. For a similar reason, the A/Cambodia strain also displayed high antibody titers, despite not being present in vaccine formulations for seasons following 2021-22. With regard to type B strains, the PrestoBlue®-based method yielded higher antibody titers than the CPE method. Of the two type B strains, B/Phuket was also found to have the highest values on applying the CPE method. Concerning this strain, the antibody titers yielded by PrestoBlue® reflect the overall picture of vaccine immunization, as B/Phuket has been present in the quadrivalent formulation since 2013, though the virus is no longer actively circulating [24].

In order to avoid false positive results, serum samples were also treated with RDE, to determine whether it was possible to eliminate the non-specific binding of serum proteins to viral antigens. It is important to note that, in most cases, RDE treatment can remove any non-specific inhibitors that could interfere with the readout [25]. Somewhat unexpectedly, RDE treatment significantly reduced the level of antibodies specific to the various influenza strains, especially subtypes A. Therefore, pre-treatment with RDE could be considered one of the fundamental steps when testing human sera, as it minimizes the risk of detecting non-specific antibodies and therefore of false positives. After treating the samples with RDE, the difference between the antibody titers obtained by means of CPE and those yielded by the colorimetric method was reduced, demonstrating that the two methods are comparable.

The use of MDCK-SIAT1 as an alternative cell substrate for H3N2 subtype influenza strains was also evaluated. As this cell line has been optimized for the growth of H3N2 influenza subtypes [26,27], we repeated titration tests in order to increase the viral titer of the strain used. However, since these cells have much longer doubling times than normal MDCK cells, it was necessary to greatly reduce the cell concentration. In any case, on using the appropriate cell line, even the A/Cambodia strain used in this study displayed titers similar to the other influenza strains used. However, our study on cell line differentiation, as well as on readout times, which appear to be strain-specific, is still ongoing.

As a final analysis in this study, the applicability of the colorimetric method was also evaluated on an avian influenza strain. In this case, too, the effectiveness of PrestoBlue® was confirmed by the comparability of the titers, thus demonstrating the objectivity of the method. In this case, however, some changes were made in terms of both the post-infection days of readout and the incubation time of PrestoBlue®. Regarding the time of readout, the ability of a virus to show its CPE is variable, even within the same family; for this reason, the method needs to be optimized according to the characteristics of the virus used. As for the incubation time of PrestoBlue®, its reduction is probably associated with the ability of the virus to infect and detach cells while still keeping them alive and therefore able to reduce the dye. This phenomenon, known as trogocytosis, has already been seen in influenza strains and, especially in strains that display extremely rapid infectivity, can result in an incorrect reading of the CPE alone, and therefore in the overestimation of the viral titer [28]. However, experiments on A/Texas are still ongoing, and further testing will be required to optimize the method.

Taken together, these data demonstrate that the colorimetric method based on the use of PrestoBlue® dye to measure antibody titers in human sera is a valid alternative to the classic method based on CPE observation with an inverted optical microscope. Its objectivity allows the raw data to be quantified and processed by means of precise mathematical formulas. Furthermore, another advantage of this method, in addition to its objectivity, is its ease of use and potential for automation, which means bypassing the need for highly specialized personnel. PrestoBlue® also serves as an alternative method to the use of specific antibodies, such as key methods that include the gold-standard Hemagglutination Inhibition (HI) assay, Microneutralization (MN) assays, and pseudotype-based neutralization (PN) assays to measure antibody-mediated viral inhibition [[29], [30], [31], [32]]. The hemagglutination inhibition assay measures the ability of antibodies to block virus particles from agglutinating red blood cells. It is a high-throughput, standardized method, though it is a surrogate for neutralization and not suitable for all influenza strains [29]. Microneutralization assay is a highly specific method that measures the ability of antibodies to prevent virus-induced cell death in MDCK cell cultures. It is considered more sensitive than HI for detecting protective antibodies [31]. The pseudotype-based neutralization assay uses safe, modified viruses (pseudotypes) expressing HA and NA instead to mimic entry, often used for high-pathogenicity strains (e.g., H5N1) to avoid strict BSL-3 laboratory requirements [32]. Some of these techniques are time-consuming as MN assays can be labor-intensive and slower than binding-based assays, reagent dependent like HI assays that require consistent sources of red blood cells and subject to escape mutants since some strains, particularly during vaccine development, can evolve to evade antibody neutralization [31,32].

The method shows the potential for higher throughput than traditional methods, enabling large-scale screening of samples to evaluate immunogenicity of new vaccines. However, the method also has limitations, such as its limited usefulness for viruses that do not produce a clear cytopathic effect and the need to use laboratories equipped for biocontainment, given the use of live viruses.

## Funding statements

This research received no external funding.

## CRediT authorship contribution statement

**Maria A. Stincarelli:** Data curation, Formal analysis, Investigation, Writing – original draft. **Francesca Dapporto:** Conceptualization, Supervision, Writing – review & editing. **Valentina Biagini:** Data curation, Formal analysis, Investigation, Writing – review & editing. **Maria Giovanna Marotta:** Data curation, Formal analysis, Investigation, Writing – review & editing. **Stefano Gonzi:** Investigation, Writing – review & editing. **Alessandro Manenti:** Methodology, Supervision, Writing – review & editing. **Emanuele Montomoli:** Methodology, Supervision, Writing – review & editing.

## Declaration of competing interest

Maria A. Stincarelli, Francesca Dapporto, Valentina Biagini, Stefano Gonzi and Alessandro Manenti are employed by VisMederi Srl. Emanuele Montomoli is an external consultant and Chief Scientific Officer of VisMederi Srl.

The authors declare that they have no known competing financial interests or personal relationships that could have appeared to influence the work reported in this paper.

## Data Availability

Data will be made available on request.

## References

[1] Jain R., Sharma H., Pena L., Jit S., Rathi B., De Oliveira R.N., Verma M. (2025). Influenza virus: genomic insights, evolution, and its clinical presentation. Microb. Pathog..

[2] Neumann G., Eisfeld A.J., Kawaoka Y. (2025). Viral factors underlying the pandemic potential of influenza viruses. Microbiol. Mol. Biol. Rev..

[3] World Helath Organization (2025). Influenza virus details. https://www.who.int/news-room/fact-sheets/detail/influenza-(seasonal).

[4] Xiong W., Zhang Z. (2025). Influenza virus genomic mutations, host barrier and cross-species transmission. Curr. Genom..

[5] Capelastegui F., Goldhill D.H. (2025). H5N1 2.3.4.4b: a review of mammalian adaptations and risk of pandemic emergence. J. Gen. Virol..

[6] De Marco M.A., Delogu M., Facchini M., Di Trani L., Boni A., Cotti C., Graziosi G., Venturini D., Regazzi D., Ravaioli V., Marzadori F., Frasnelli M., Castrucci M.R., Raffini E. (2021). Serologic evidence of occupational exposure to Avian influenza viruses at the wildfowl/poultry/human interface. Microorganisms.

[7] Torres A., Cilloniz C., Aldea M., Mena G., Miró J.M., Trilla A., Vilella A., Menéndez R. (2025). Adult vaccinations against respiratory infections. Expert Rev. Anti Infect. Therapy.

[8] Hendin H.E., Lavoie P.O., Gravett J.M., Pillet S., Saxena P., Landry N., D'Aoust M.A., Ward B.J. (2022). Elimination of receptor binding by influenza hemagglutinin improves vaccine-induced immunity. NPJ Vaccines.

[9] Myburgh L., Van Loon K., Huijbers E.J.M., Van Beijnum J.R., Russell C.A., Griffioen A.W. (2025). Guided design for the development of an evolution-proof influenza vaccine. Vaccine.

[10] Steventon R., Stolle L., Thompson C.P. (2025). How broadly neutralising antibodies are redefining immunity to influenza. Antibodies.

[11] Sun X., Ling Z., Yang Z., Sun B. (2022). Broad neutralizing antibody-based strategies to tackle influenza. Curr. Opin. Virol..

[12] Zhang R., Hung I.F. (2021). Approaches in broadening the neutralizing antibody response of the influenza vaccine. Expert Rev. Vaccine.

[13] Creanga A., Gillespie R.A., Fisher B.E., Andrews S.F., Lederhofer J., Yap C., Hatch L., Stephens T., Tsybovsky Y., Crank M.C., Ledgerwood J.E., McDermott A.B., Mascola J.R., Graham B.S., Kanekiyo M. (2021). A comprehensive influenza reporter virus panel for high-throughput deep profiling of neutralizing antibodies. Nat. Commun..

[14] Calzado-Dacasin C., Foronda J.L., Arguelles V.L., Daga C.M., Quimpo M.T., Lupisan S., Dapat C., Saito M., Okamoto M., Albano P.M., Oshitani H. (2022). Serotype identification of human adenoviruses associated with influenza-like illnesses in the Philippines from 2006-2012 by microneutralization and molecular techniques. Int. J. Infect. Dis..

[15] Miller P.D.E., de Silva T.I., Leonard H., Anthias C., Hoschler K., Goddard K., Peggs K., Madrigal A., Snowden J.A. (2019). A comparison of viral microneutralization and haemagglutination inhibition assays as measures of seasonal inactivated influenza vaccine immunogenicity in the first year after reduced intensity conditioning, lymphocyte depleted allogeneic haematopoietic stem cell transplant. Vaccine.

[16] Waldock J., Weiss C.D., Wang W., Levine M.Z., Jefferson S.N., Ho S., Hoschler K., Londt B.Z., Masat E., Carolan L., Sanchez-Ovando S., Fox A., Watanabe S., Akimoto M., Sato A., Kishida N., Buys A., Maake L., Fourie C., Caillet C., Raynaud S., Webby R.J., DeBeauchamp J., Cox R.J., Lartey S.L., Trombetta C.M., Marchi S., Montomoli E., Sanz-Muñoz I., Eiros J.M., Sanchez-Martınez J., Duijsings D., Engelhardt O.G. (2023). An external quality assessment feasibility study; cross laboratory comparison of haemagglutination inhibition assay and microneutralization assay performance for seasonal influenza serology testing: a FLUCOP study. Front. Immunol..

[17] Nath Neerukonda S., Vassell R., Weiss C.D. (2020). Neutralizing antibodies targeting the conserved stem region of influenza hemagglutinin. Vaccines (Basel).

[18] Abo Shama N.M., Mahmoud S.H., Bagato O., AbdElsalam E.T., Alkhazindar M., Kandeil A., McKenzie P.P., Webby R.J., Ali M.A., Kayali G., El-Shesheny R. (2022). Incidence and neutralizing antibody seroprevalence of influenza B virus in Egypt: results of a community-based cohort study. PLoS One.

[19] Ilyushina N.A., Komatsu T.E., Ince W.L., Donaldson E.F., Lee N., O'Rear J.J., Donnelly R.P. (2019). Influenza A virus hemagglutinin mutations associated with use of neuraminidase inhibitors correlate with decreased inhibition by anti-influenza antibodies. Virol. J..

[20] Liang W., Lv H., Chen C., Sun Y., Hui D.S., Mok C.K.P. (2023). Lack of neutralizing antibodies against influenza A viruses in adults during the 2022/2023 winter season - a serological study using retrospective samples collected in Hong Kong. Int. J. Infect. Dis..

[21] Li J., Zhang L., Bao L., Wang Y., Qiu L., Hu J., Tang R., Yu H., Shan J., Li Y., Qin C., Zhu F. (2022). A broadly neutralizing human monoclonal antibody against the hemagglutinin of avian influenza virus H7N9. Chin. Med. J..

[22] Manenti A., Maggetti M., Casa E., Martinuzzi D., Torelli A., Trombetta C.M., Marchi S., Montomoli E. (2020). Evaluation of SARS-CoV-2 neutralizing antibodies using a CPE-based colorimetric live virus micro-neutralization assay in human serum samples. J. Med. Virol..

[23] Zhong S., Ng T.W.Y., Skowronski D.M., Iuliano A.D., Leung N.H.L., Perera R.A.P.M., Ho F., Fang V.J., Tam Y.H., Ip D.K.M., Havers F.G., Fry A.M., Aziz-Baumgartner E., Barr I.G., Peiris M., Thompson M.G., Cowling B.J. (2024). Influenza A(H3N2) antibody responses to standard-dose versus enhanced influenza vaccine immunogenicity in older adults and prior season's vaccine status. JID (J. Infect. Dis.).

[24] World Helath Organization (2025). Recommendations for influenza vaccine composition. https://www.who.int/teams/global-influenza-programme/vaccines/who-recommendations.

[25] Goshina A., Matyushenko V., Mezhenskaya D., Rak A., Katelnikova A., Gusev D., Rudenko L., Isakova-Sivak I. (2023). RDE treatment prevents non-specific detection of SARS-CoV-2- and influenza-specific IgG antibodies in heat-inactivated serum samples. Antibodies.

[26] Aw D.Z.H., Heng K.K., Heok J.Y.H., Kong X.Y., Chen H., Zhang T., Zhai W., Chow V.T.K. (2022). Serial passaging of seasonal H3N2 influenza A/Singapore/G2-31.1/2014 virus in MDCK-SIAT1 cells and primary chick embryo cells generates HA D457G mutation and other variants in HA, NA, PB1, PB1-F2, and NS1. Int. J. Mol. Sci..

[27] Matsumoto S., Chong Y., Kang D., Ikematsu H. (2019). High genetic stability in MDCK-SIAT1 passaged human influenza viruses. J. Infect. Chemother..

[28] Kongsomros S., Manopwisedjaroen S., Chaopreecha J., Wang S.F., Borwornpinyo S., Thitithanyanont A. (2021). Rapid and efficient cell-to-cell transmission of Avian influenza H5N1 virus in MDCK cells is achieved by trogocytosis. Pathogens.

[29] Chen B., Zambrana J.V., Shotwell A., Sanchez N., Plazaola M., Ojeda S., Lopez R., Stadlbauer D., Kuan G., Balmaseda A., Krammer F., Gordon A. (2026). Hemagglutination inhibition and alternate serologic responses following influenza A(H3N2) virus. Infection.

[30] Martins A.M., Valero Juan L.F., Santos M., Martins J.P. (2025). Immunogenicity as a predictor of influenza vaccine efficacy: a systematic review. Vaccines (Basel).

[31] Azeem S., Yoon K.J. (2025). Diagnostic assays for Avian influenza virus surveillance and monitoring in poultry. Viruses.

[32] Tsai C., Caillet C., Hu H., Zhou F., Ding H., Zhang G., Zhou B., Wang S., Lu S., Buchy P., Deubel V., Vogel F.R., Zhou P. (2009). Measurement of neutralizing antibody responses against H5N1 clades in immunized mice and ferrets using pseudotypes expressing influenza hemagglutinin and neuraminidase. Vaccine.

